# Assessment of Quality of Life in Patients With End-Stage Kidney Disease on Maintenance Hemodialysis Using the Missoula-Vitas Quality of Life Index

**DOI:** 10.7759/cureus.65459

**Published:** 2024-07-26

**Authors:** Yahya Azeem Ahmed, Iqra Naeem, Samee Javed Bhatti, Badar U Din Shah, Adnan Ahmad Zafar, Adeel Ahmed, Muhammad Irfan Jamil

**Affiliations:** 1 Nephrology, Nishtar Medical University, Multan, PAK; 2 Psychiatry and Behavioral Sciences, Lahore General Hospital, Lahore, PAK; 3 Nephrology, Lahore General Hospital, Lahore, PAK; 4 Nephrology, Geisinger Medical Center, Danville, USA; 5 Nephrology, Islam Medical College Sialkot, Lahore, PAK; 6 Medicine, Lahore General Hospital, Lahore, PAK

**Keywords:** maintenance hemodialysis, missoula-vitas quality of life index, quality-of-life, chronic kidney disease (ckd), end stage renal disease (esrd)

## Abstract

Aim and background: This study aimed to evaluate the quality of life (QoL) in end-stage kidney disease (ESRD) patients on maintenance hemodialysis through the Missoula-Vitas Quality of Life Index-15 (MVQOLI-15) to identify factors affecting their well-being.

Materials and methods: A cross-sectional study was conducted at the Dialysis Unit of the Nephrology Department, Nishtar Hospital Multan. Over six months, 140 eligible patients were enrolled using non-probability consecutive sampling. Participants aged 18-80 years on maintenance hemodialysis for at least six months were evaluated using the MVQOLI-15 questionnaire assessing symptoms, function, interpersonal, well-being, and transcendence dimensions of QoL. Data were analyzed using the IBM SPSS Statistics for Windows, Version 26 (Released 2019; IBM Corp., Armonk, New York). Inferential statistical tests, including the t-test for comparing two groups and analysis of variance (ANOVA) for comparing multiple groups, were utilized to determine the significance of differences in QoL scores among different demographic and clinical categories. P-values less than 0.05 were considered statistically significant.

Results: The study analyzed 140 hemodialysis patients, with a mean age of 52.41 ± 16.31 years and an average hemodialysis duration of 4.55 ± 2.46 years. Most participants were aged 61-80 years (35.7%), had secondary education (44.3%), and were married (67.1%). QoL scores, measured using the MVQOLI, indicated mean values for symptoms at 4.51 ± 10.71, function at 5.77 ± 8.04, interpersonal at 7.49 ± 13.67, well-being at -13.60 ± 7.11, transcendence at 8.24 ± 13.12, and a total score of 16.24 ± 2.75. Significant findings include the following: females had higher symptom scores (p=0.001) and lower well-being scores (p=0.000); younger patients (<30 years) had higher function scores (p=0.054); patients on hemodialysis three times per week had higher function scores (p=0.006); patients taking 1 to 3 pills per day had higher transcendence scores (p=0.000); unmarried patients had higher symptoms scores (p=0.064) and lower well-being scores (p=0.004); and illiterate patients had higher symptoms (p=0.005) and transcendence scores (p=0.034). In total score, patients on hemodialysis once per week reported significantly better scores (p=0.011).

Conclusion: This study highlights varied QoL experiences among hemodialysis patients, with transcendence scoring the highest and well-being, the lowest. Demographic factors such as age, gender, and education level significantly impact the QoL dimensions. Understanding these findings can guide personalized interventions to improve the well-being of hemodialysis patients.

## Introduction

Chronic kidney disease (CKD) is a major public health concern globally, with a prevalence of 13.4% (11.7-15.1%) [1]. Patients with end-stage kidney disease (ESKD), the final stage of CKD, often require renal replacement therapy such as dialysis or kidney transplantation. Approximately 4.9 to 7 million individuals worldwide are affected by ESKD [2]. ESKD significantly impacts patients' quality of life (QoL) due to impairments and limitations across various aspects of daily living. These include physical, emotional, and social challenges, necessitating comprehensive care and management strategies to improve patient outcomes [2].

Hemodialysis is a demanding treatment that causes major interruptions to patients' daily lives because it requires them to visit hospitals or dialysis facilities regularly, usually two to three times a week [3]. Several studies have reported that patients on hemodialysis (HD) are less physically active and have lower exercise capacity and poorer physical functioning than persons with normal kidney function [4,5]. Patients on hemodialysis have reduced QoL scores compared to those with normal kidney function. The morbidity and mortality rates of HD patients are directly related to their QoL [6]. Many patients undergoing long-term hemodialysis experience social isolation and self-isolation from society, a state known as “social death.” This issue underscores the need for effective social adaptation and re-socialization strategies [7]. In clinical practice, assessing QoL is essential for managing chronic diseases. Various questionnaires and tools are currently available to assess the severity of social, physical, and psycho-social adaptations in patients on long-term hemodialysis [8].

The Missoula-Vitas Quality of Life Index (MVQOLI) scale was created and validated in the United States by Byock and Merriman to assess QoL in patients with advanced illnesses within palliative care settings [9]. Maintaining optimal QoL is an important goal of palliative care, and data gathered via the MVQOLI-15 questionnaire help healthcare professionals identify and address patient concerns that impact QoL [10]. This study aimed to utilize the MVQOLI to assess patients' experiences across multiple aspects. By examining symptoms, functionality, relationships, well-being, and transcendence, we can gain valuable insights into the challenges faced by patients on maintenance hemodialysis. The findings will inform healthcare providers about key areas of concern, seeking to improve the overall QoL and care delivery for hemodialysis patients.

## Materials and methods

A cross-sectional study was conducted at the Dialysis Unit of the Nephrology Department of Nishtar Hospital Multan, after obtaining ethical approval from the Institutional Review Board (IRB No. 7510). The study took place over six months, from December 2023 to May 2024. From the initial pool of 350 patients receiving maintenance hemodialysis at our center, 140 participants were enrolled in the study using non-probability consecutive sampling after applying the eligibility criteria and obtaining informed consent.

This study included both male and female end-stage kidney disease patients on maintenance hemodialysis for at least six months, aged between 18 and 80 years, who showed cooperation and had no language barriers. Patients who were blind or severely visually impaired, experienced complications such as hypoglycemia or hypotension during hemodialysis, were seriously ill, or were unwilling to participate were excluded from the study. Additionally, patients with dementia or cognitive impairment were also excluded. Patients were asked for sociodemographic and clinical data, such as age, gender, level of education, marital status, social status, length and frequency of hemodialysis, daily prescription pill count, and MVQOLI-15 score. A trained doctor conducted the questionnaire by asking the questions directly to the participants. Patients were assured that all information would remain confidential.

The MVQOLI-15 scale validated Urdu version was used for QoL assessment of patients. The questionnaire covered five dimensions: Symptoms, Function, Interpersonal, Well-Being, and Transcendence. This questionnaire was completed in 30 minutes. In each dimension, three types of information were collected: Assessment, Satisfaction, and Importance. Assessment questions were rated on a five-point Likert scale from -2 to +2, Satisfaction questions from -4 to +4, and Importance questions from 1 to 5. To calculate the overall score for each dimension, the Assessment (A) and Satisfaction (S) scores were summed and then multiplied by the Importance (I) score: (A + S) x I. Each dimension score ranged from -30 to +30, reflecting its impact on overall QoL. The total MVQOLI-15 score was calculated using the formula: sum of dimension scores/10 + 15, resulting in a QoL rating scale ranging from 0 to 30. Higher total scores indicated a higher level of QoL [10].

Data were analyzed using the IBM SPSS Statistics for Windows, Version 26 (Released 2019; IBM Corp., Armonk, New York). Categorical variables were presented as frequency and percentages. Descriptive statistics, such as means and standard deviations, were calculated to summarize the QoL scores across different groups. Inferential statistical tests, including the t-test for comparing two groups and analysis of variance (ANOVA) for comparing multiple groups, were utilized to determine the significance of differences in QoL scores among different demographic and clinical categories. P-values were calculated to assess the statistical significance of these differences, with values less than 0.05 considered statistically significant.

## Results

The mean age of participants was 52.41 ± 16.31 years. The average duration of hemodialysis was 4.55 ± 2.46 years (Table 1).

**Table 1:** Demographic and clinical characteristics of study participants. HD: hemodialysis.

Demographic and clinical characteristics	Frequency	Percentage
Age groups		
<30 years	18	12.9
31 to 45 years	30	21.4
46 to 60 years	42	30.0
61 to 80 years	50	35.7
Gender		
Male	70	50.0
Female	70	50.0
Educational status		
Illiterate	28	20.0
Primary	40	28.6
Secondary	62	44.3
Higher	10	7.1
Marital status		
Unmarried	20	14.3
Married	94	67.1
Divorced	26	18.6
Social status		
Unemployed	44	31.4
Employed	68	48.6
Retired	28	20.0
Duration of HD		
<1 year	12	8.6
1 to 5 years	70	50.0
>5 years	58	41.4
Frequency of HD		
1/week	50	35.7
2/week	80	57.1
3/week	10	7.1
Number of pills per day		
1 to 3	22	15.7
4 to 7	68	48.6
>7	50	35.7

QoL scores measured by MVQOLI showed the following means and standard deviations: symptom score 4.51 ± 10.71, function score 5.77 ± 8.04, interpersonal score 7.49 ± 13.67, well-being score -13.60 ± 7.11, transcendence score 8.24 ± 13.12, and total score 16.24 ± 2.75. The QoL of hemodialysis patients was analyzed based on various demographic and clinical variables.

Notably, the highest transcendence score was observed in males (10.23 ± 13.20, p=0.073). In contrast, females showed a significantly higher symptom score (7.49 ± 10.53, p=0.001). Patients aged <30 years exhibited the highest function score (9.56 ± 7.64, p=0.054). Patients taking one to three pills per day had a significantly higher transcendence score (18.55 ± 8.05, p=0.000). Patients undergoing hemodialysis three times per week demonstrated the highest function score (11.40 ± 5.60, p=0.006). Additionally, those with a duration of hemodialysis of less than one year showed the highest function score (9.83 ± 2.98, p=0.061). Unemployed patients had the highest transcendence score (8.73 ± 13.73, p=0.940), and unmarried patients had a higher symptom score (9.60 ± 9.13, p=0.064), both of which were not statistically significant. The highest symptom score was observed in illiterate patients (9.79 ± 9.11, p=0.005), which was significant. Significant findings also included the lowest well-being score observed in females (-16.03 ± 7.42, p=0.000) and patients taking one to three pills per day (-16.91 ± 8.02, p=0.046) (Table 2).

**Table 2:** Comparison between demographic and clinical characteristics and Missoula-Vitas Quality of Life Index parameters in hemodialysis patients.

Category	Symptoms (mean ± SD)	Function (mean ± SD)	Interpersonal (mean ± SD)	Well-being (mean ± SD)	Transcendence (mean ± SD)	Total score (mean ± SD)
Gender						
Male	1.54 ± 10.12	5.49 ± 7.10	8.57 ± 13.14	-11.17 ± 5.89	10.23 ± 13.20	16.47 ± 2.74
Female	7.49 ± 10.53	6.06 ± 8.93	6.40 ± 14.19	-16.03 ± 7.42	6.26 ± 12.83	16.02 ± 2.76
p-value	0.001	0.676	0.349	0.000	0.073	0.337
Age group (years)						
<30	6.22 ± 10.80	9.56 ± 7.64	6.67 ± 17.48	-17.00 ± 6.38	8.67 ± 9.55	16.41 ± 2.24
31 to 45	4.40 ± 10.52	4.13 ± 5.30	12.67 ± 12.84	-15.13 ± 8.20	9.93 ± 11.38	16.60 ± 2.64
46 to 60	4.95 ± 10.07	4.19 ± 10.07	6.67 ± 13.42	-14.05 ± 7.66	9.14 ± 13.12	16.09 ± 2.70
61 to 80	3.60 ± 11.51	6.72 ± 7.19	5.36 ± 12.40	-11.08 ± 5.28	6.32 ± 15.16	16.09 ± 3.07
p-value	0.830	0.054	0.124	0.006	0.622	0.840
Number of pills per day						
1 to 3 pills	4.00 ± 7.83	5.55 ± 6.43	6.18 ± 14.39	-16.91 ± 8.02	18.55 ± 8.05	16.74 ± 2.12
4 to 7 pills	5.18 ± 11.91	7.06 ± 6.38	6.50 ± 12.95	-13.35 ± 5.76	6.62 ± 13.90	16.20 ± 2.78
>7 pills	3.84 ± 10.20	4.12 ± 10.24	9.40 ± 14.35	-12.48 ± 8.01	5.92 ± 11.81	16.08 ± 2.98
p-value	0.778	0.145	0.467	0.046	0.000	0.641
Frequency of hemodialysis						
1/week	5.88 ± 9.85	7.28 ± 5.76	10.08 ± 12.89	-14.16 ± 7.60	11.24 ± 11.24	17.03 ± 2.38
2/week	3.52 ± 11.52	4.12 ± 9.02	6.00 ± 14.24	-12.83 ± 6.70	5.58 ± 14.09	15.64 ± 2.95
3/week	5.60 ± 7.68	11.40 ± 5.60	6.40 ± 11.94	-17.00 ± 7.18	14.60 ± 8.47	17.10 ± 1.56
p-value	0.453	0.006	0.247	0.170	0.015	0.011
Duration of hemodialysis						
<1 year	3.67 ± 9.47	9.83 ± 2.98	8.83 ± 18.85	-17.67 ± 3.98	9.83 ± 6.91	16.45 ± 2.46
1 to 5 years	3.49 ± 10.63	4.43 ± 9.21	6.46 ± 14.62	-12.91 ± 7.62	7.14 ± 13.44	15.86 ± 2.92
>5 years	5.93 ± 11.06	6.55 ± 6.85	8.45 ± 11.21	-13.59 ± 6.76	9.24 ± 13.75	16.66 ± 2.58
p-value	0.423	0.061	0.673	0.101	0.609	0.255
Social status						
Unemployed	5.27 ± 11.37	4.59 ± 9.01	5.86 ± 13.87	-13.64 ± 7.21	8.73 ± 13.73	16.08 ± 3.02
Employed	4.59 ± 10.40	5.65 ± 8.22	8.35 ± 14.63	-14.32 ± 7.96	7.85 ± 12.08	16.21 ± 2.86
Retired	3.14 ± 10.66	7.93 ± 5.35	7.93 ± 10.86	-11.79 ± 3.89	8.43 ± 14.93	16.56 ± 2.02
p-value	0.714	0.227	0.634	0.284	0.940	0.766
Marital status						
Unmarried	9.60 ± 9.13	8.70 ± 7.05	11.00 ± 10.65	-18.40 ± 6.33	7.10 ± 9.07	16.80 ± 1.12
Married	3.89 ± 10.63	5.28 ± 8.26	7.40 ± 14.00	-12.68 ± 6.98	8.09 ± 12.54	16.20 ± 2.80
Divorced	2.85 ± 11.35	5.31 ± 7.74	5.08 ± 14.40	-13.23 ± 6.88	9.69 ± 17.50	15.97 ± 3.41
p-value	0.064	0.214	0.347	0.004	0.788	0.580
Educational status						
Illiterate	9.79 ± 9.11	5.36 ± 7.37	5.21 ± 14.74	-12.43 ± 5.23	8.57 ± 9.45	16.65 ± 1.91
Primary	4.30 ± 10.88	3.75 ± 9.95	8.35 ± 10.23	-14.60 ± 8.23	8.35 ± 11.69	16.02 ± 2.38
Secondary	1.65 ± 10.01	7.74 ± 6.42	7.65 ± 14.77	-13.55 ± 7.03	9.87 ± 14.79	16.34 ± 3.04
Higher	8.40 ± 13.07	2.80 ± 8.52	9.40 ± 16.63	-13.20 ± 7.81	-3.20 ± 12.35	15.42 ± 4.18
p-value	0.005	0.051	0.772	0.667	0.034	0.609

## Discussion

The results of this study revealed some unexpected findings. Contrary to the initial hypothesis that patients would report low QoL scores due to the significant challenges posed by their disease and treatment, the study participants reported relatively high QoL scores across most dimensions, with the exception of well-being. This positive assessment suggests an increased perceived QoL among the study population. The mean age of patients in this study was 52.41 ± 16.31 years, which is slightly lower than the mean ages reported by Dimova et al. at 57.6 ± 14.0 years and Gerasimoula et al. at 60.2 ± 11.8 years [3,7]. The total MVQOLI score in this study was 16.24 ± 2.75, comparable to scores reported in other studies: Dimova et al. found 16.44 ± 4.12, Gerasimoula et al. reported 17.43 ± 4.76, and Theofilou et al. observed 17.36 ± 3.76 [3,7,11].

Unlike previous studies, where the highest score was observed in the interpersonal dimension (Gerasimoula et al. at 8.20 ± 1.64, Dimova et al. at 8.23 ± 16.31), this study reported the highest score in the transcendence parameter (8.24 ± 13.12) [3,7]. Consistent with previous findings, this study also reported the lowest score in the well-being dimension (-13.60 ± 7.11). Our results revealed that male participants experienced a higher QoL compared to females. This outcome aligns with previous research indicating that men undergoing hemodialysis often report better QoL scores than women [3,5]. Our study found that patients aged 31 to 45 years had a function score of 4.13 ± 5.30, while those aged below 30 years had a higher score of 9.56 ± 7.64, aligning with Gerasimoula et al.'s findings. Both studies indicate younger hemodialysis patients tend to report better QoL [3]. However, another study reported superior QoL among older patients [7]. Gender and age inconsistencies in perceived QoL persist, with males and younger patients generally reporting better outcomes. These results underline the need for targeted interventions addressing specific demographic groups and QoL in hemodialysis care.

In agreement with Al Zahrani et al., our study reported that patients undergoing dialysis once per week had better QoL than those receiving dialysis two or three times per week [1]. In our study, only those patients receiving hemodialysis once weekly had significant renal reserves that allowed them to tolerate uremic symptoms even on a single dialysis per week. Contrarily, most of the previous literature reported that frequent dialysis improves QoL [4,12]. Additionally, patients on hemodialysis for less than one year reported better scores in all domains than those on treatment for more than one year. This observation demonstrates that as the disease becomes more chronic, the QoL becomes increasingly compromised due to dialysis-related complications that patients experience over time [13]. Contrary to common belief and results reported by Gerasimoula et al., our study observed that patients with lower education levels reported better scores across MVQOLI-15 dimensions [3]. This unexpected result suggests that higher education might lead to more critical evaluation of care and treatment satisfaction. These findings align with previous research, challenging perceptions about education's impact on QoL in hemodialysis patients [1,14]. Our study and Al Zahrani et al. revealed that retired patients reported better QoL compared to those who were employed, while divorced individuals experienced lower QoL than those who were married or single [1].

The limitations of this study include a small sample size, recruitment from a single center, and targeting a specific demographic, which may restrict the generalizability of the results. The QoL assessments were conducted during hemodialysis sessions, potentially affecting focus and responses. However, the study's strengths include a well-defined, homogeneous target population and the use of a standardized QoL questionnaire. Future research should involve larger, multicenter studies to confirm these results and evaluate interventions aimed at enhancing QoL for hemodialysis patients. Future studies should conduct baseline assessments, implement targeted education and psychological support for hemodialysis patients, perform post-intervention evaluations, and explore longitudinal changes in QoL to enhance patient care strategies.

## Conclusions

This research highlights the complex interplay of factors affecting the QoL in hemodialysis patients. Transcendence emerged as a strength, while well-being remains a critical area for improvement. Younger patients, males, and those with lower education levels unexpectedly reported better outcomes in certain dimensions. Treatment frequency and duration also influenced QoL scores. These insights emphasize the importance of personalized approaches in hemodialysis care strategies to address the diverse QoL challenges faced by hemodialysis patients.
